# Spot the Difference: Mimicry in a Coral Reef Fish

**DOI:** 10.1371/journal.pone.0055938

**Published:** 2013-02-13

**Authors:** Monica Gagliano, Martial Depczynski

**Affiliations:** 1 Centre for Evolutionary Biology, School of Animal Biology, University of Western Australia, Crawley, Western Australia, Australia; 2 Centre for Microscopy, Characterisation and Analysis, University of Western Australia, Crawley, Western Australia, Australia; 3 Australian Institute of Marine Science, The Oceans Institute, University of Western Australia, Crawley, Western Australia, Australia; Emory University, United States of America

## Abstract

Eyespots on the body of many animals have long been assumed to confer protection against predators, but empirical evidence has recently demonstrated that this may not always be the case and suggested that such markings may also serve other purposes. Clearly, this raises the unresolved question of what functions do these markings have and do they contribute to an individual’s evolutionary fitness in the wild. Here, we examined the occurrence of eyespots on the dorsal fin of a coral reef damselfish (*Pomacentrus amboinensis*), where these markings are typical of the juvenile stage and fade away as the fish approaches sexual maturation to then disappear completely in the vast majority of, but not all, adult individuals. By exploring differences in body shape among age and gender groups, we found that individuals retaining the eyespot into adulthood are all sexually mature males, suggesting that these eyespots may be an adult deceptive signal. Interestingly, the body shape of these individuals resembled more closely that of immature females than mature dominant males. These results suggest that eyespots have multiple roles and their functional significance changes within the lifetime of an animal from being a juvenile advertisement to a deceptive adult signal. Male removal experiments or colour manipulations may be necessary to establish specific functions.

## Introduction

The widespread occurrence and adaptive significance of eyespots on the body of many animals have long intrigued biologists. Current understanding strongly promotes the paradigm that these conspicuous eye-resembling colour markings have various antipredatory functions, such as deterring hunting predators to initiate an attack by resembling the eyes of their predator’s own enemies (i.e. intimidation hypothesis) or diverting their attacks toward less vital body parts (i.e. deflective hypothesis [1]). A range of studies have indeed provided good evidence for an intimidatory and startling function of eyespots in insects such as peacock butterflies [2] and eyed hawk moths [3], and even artificial prey [4], [5], showing that these eyespots can be highly effective in scaring birds. On the other hand, studies of deflective effects generally ascribed to peripheral eyespots located at the posterior end of the body far from the animal’s head (e.g. butterflies [6]; fishes [7] and frogs [8]) have not found any convincing support ([9]–[11] but see [12]–[13]). If these eyespots are not aimed at deflecting attacking predators, then what function (if any) do they really serve and does it contribute to an individual’s evolutionary fitness? The function of eyespots in a non-predatory role has been previously investigated, specifically in the context of mate choice in the butterfly *Bicyclus anynana* [14]–[16]. Yet, other than in systems with Lepidoptera and avian or lizard predators, investigations of the alternative, non-predatory value of eyespots are relatively rare. Empirical evidence supporting the idea that these markings may have a different function and that they may be important in reproduction has only recently been provided in fishes [11].

Eyespots of the coral reef damselfish *Pomacentrus amboinensis* have been suggested to serve as a subordinate signal directed at dominant males [11]. They consist of a large black spot surrounded by a brilliant white ring found on the dorsal fin of all juvenile individuals and, similar to the sub-adult plumage of young birds, may be a form of status signalling (i.e. status signalling hypothesis [17]) relaying an honest signal of social sub-ordinance to adult territorial males. Because *P. ambionensis* forms social groups of individuals of all ages, where multiple mature and immature females are loosely centred around a dominant male [18], the main advantage of being accepted within defended male territories is known to be a reduced risk of predation (e.g. survival rates within male territories are approximately 4 times higher than in adjacent areas [19]). In addition, because males show little aggression towards recruit-stage conspecifics, the advantages of living in defended territories may extend to increased access and availability of food and habitat resources, resulting from lower densities of competitive or aggressive species. Being a protogynous species, all *P. amboinensis* juveniles start out as females and interestingly, their eyespots fade as the fish approaches sexual maturation to disappear completely in the vast majority of mature adult individuals, but intriguingly, not all.

For those adults that retain their dorsal eyespots, we hypothesized that the presence of the eyespot may be preventing dominant males with territories from distinguishing “bluffing” mature males in juvenile (female) clothing from truly sexually immature female individuals in order to go unrecognized as competing and fertile “sneaker” males (i.e. dual male reproductive strategies [20]–[23]). Because cheats are supposed to go unnoticed by sharing common perceived characteristics of those being mimicked, clear cases of dishonest signalling, where certain individuals mask their true identity by taking on, for example, the appearance of females (e.g. female mimicry [24]) may be generally difficult to detect. While the adaptive significance of signals used in mimicry is often examined in terms of colouration and colour patterns of individuals, behavior and body shape (or a combination of them) can also have a signal function and be mimicked by others [25]. In fishes, subtle variations in body shape can reveal important ecological and behavioural differences [26], and hence provide useful information for improving our understanding of animal signals and their relevance in the evolution of animal social systems. Hence, the overall aim of this study was to examine the possible functional role of the dorsal eyespots in *P. amboinensis* by exploring differences in body shape among age and gender groups. Because mature adults are not sexually dichromatic (aside from the retention or disappearance of the eyespot) and all individuals mature first as females and later change sex to function as males (i.e. monandric protogynous hermaphroditism [27]), this model species and system provide an ideal opportunity for examining ontogenetic changes in eyespot function within a socio-behavioural context. Specifically, we first tested whether larger individuals retaining the eyespot are in fact all sexually mature individuals, and exclusively males. We then asked (1) whether the body shape of eye-spotted adults more closely resembles that of juveniles and immature females (i.e. mimicry) than that of mature males; and if so, (2) whether such variation in reproductive tactics is age-dependent (e.g. individuals may be sneakers when young and become parentals when older).

## Materials and Methods

### Ethics Statement

The study was conducted in accordance with the James Cook University Animal Ethics guidelines (Permit Number: A-1254) and under appropriate permits from the Great Barrier Reef Marine Park Authority.

The study was conducted during the austral summer at Lizard Island (14° 38′S, 145° 28′E) on the northern Great Barrier Reef. During the breeding season, two divers used hand nets to randomly collect a total of 113 *P. amboinensis* of various sizes and stages of maturation from a contiguous reef. Each fish was placed in an individually numbered transparent plastic bag and sexed *in situ* by visual inspection of its genital papillae [28] by both divers to assign it to 1 of 4 groups: [j] true juveniles (i.e. recently settled individuals; less than 3 months old), [f] immature females (i.e. 3–6 months old juvenile), [F] mature females or [M] mature males. Each individual was then photographed in side view against a measuring board with 1-cm gradations as a scale for subsequent image calibration and immediately released. A small subsample of 25 individuals was retained for dissection in the laboratory to assess the maturation state of their gonads (i.e. immature vs. developed testes/ovaries). This examination indicated that our field scores were 96% correct in assigning individuals to a specific group, hence making the visual inspection method used here a reliable and ethically sound approach.

Digital images for body shape analyses were taken and each individual scored based on the presence/absence of a dorsal fin eyespot. All images were calibrated using the scale on the photograph itself and the dimensions of each fish quantified using the image analysis program OPTIMAS 6.5. Fast Fourier analysis was then used to describe the overall body shape of the fish as previously done in similar studies [29]. Briefly, measurements (mm) of standard length (SL), depth of body 1 (DB_1_) measured from the anterior base of dorsal fin to the anterior base of pelvic fin, and depth of body 2 (DB_2_) measured across the body at the anterior base of anal fin were taken. Secondly, the outline of the body excluding all fins and across the base of the caudal peduncle was then traced to estimate perimeter (P), area (A mm^2^) and rectangularity (R, where R = A/SL x DB_1_), an index calculating how well the body shape fits into a rectangular shape. Lastly, the silhouette of each fish was described by sampling over 128 equidistant points along the same body outline, extracting a series of successive cosine waves, having phase angle and amplitude components. Hence, the outline of the body was produced as an aggregate of simple waveforms, where the amplitude of each cosine wave defined a Fast Fourier shape descriptor (also called ‘harmonic’ [H*n*]). By setting the zero^th^ descriptor (H_0_) to 0+0*_i_*, we standardize all successive harmonics to account for differences in the position of the body silhouette on the screen; we then divided all successive harmonics by H_1_ to remove any confounding effect of body size from the data. Because each descriptor produces a simplified representation of the original shape and thus reproduces only some aspects of it, multiple descriptors contributing to increasingly finer details of the original shape may be necessary to entirely reconstruct the complete outline. Accordingly, the first six harmonics (H_2_–H_6_) are referred to as low-order descriptors and determine the gross shape of the body such as its elongation, triangularity and squareness, whereas successively higher descriptors measure increasingly finer details of the body outline. In this study, the number of harmonics to be used as shape descriptors for each fish was set to the first 20 (excluding H_0_ and H_1_) because the contribution of higher order harmonics (i.e. 22 and above) to the definition of the shape was negligible [29].

All data were checked for and met the requirements of normality and homogeneity of variance before performing statistical analyses. One-way ANOVAs were used to compare morphological measures (standardized by standard length) of individuals at different stages of maturation and with/without an eyespot on their dorsal fin. All differences among treatments were identified using post-hoc Tukey’s [honest significant difference (HSD)] tests. To quantify the precision of our measures, randomly selected digital images of 10 individuals were re-calibrated twice on separate occasions and re-measured. The overall errors associated with obtaining measurements from the digital images were very low [e.g. coefficient of variation (CV) values: SL, 2.91%; BD_1_, 3.07%; BD_2_, 3.57%, P, 1.98%; A, 2.52%; R, 1.31%], indicating that the morphological data were collected and quantified reliably.

Using the first 20 standardized harmonics (H_2_–H_21_), the body shape of all photographed *P. amboinensis* were compared in relation to their maturation status and the presence/absence of an eyespot. The hypothesis of no difference in body shape among the groups was tested using multivariate analysis of variance (MANOVA), followed by a canonical discriminant analysis (CDA) to examine and display the patterns of difference identified by MANOVA. Vectors of the original harmonics were plotted to aid interpretation of differences among groups. The length of the vectors described the relative importance of each harmonic in discriminating among groups. Each group was represented by 95% confidence cloud around group centroids.

To provide further information on the age of *P. amboinensis* with and without eyespots, we examined the information stored within the otoliths of a total of 80 individuals. Transverse sections of sagittal otoliths were obtained by mounting individual otoliths in thermoplastic cement and the daily increments visible from these sections used to estimate age [30].

## Results and Discussion

**Figure 1:**
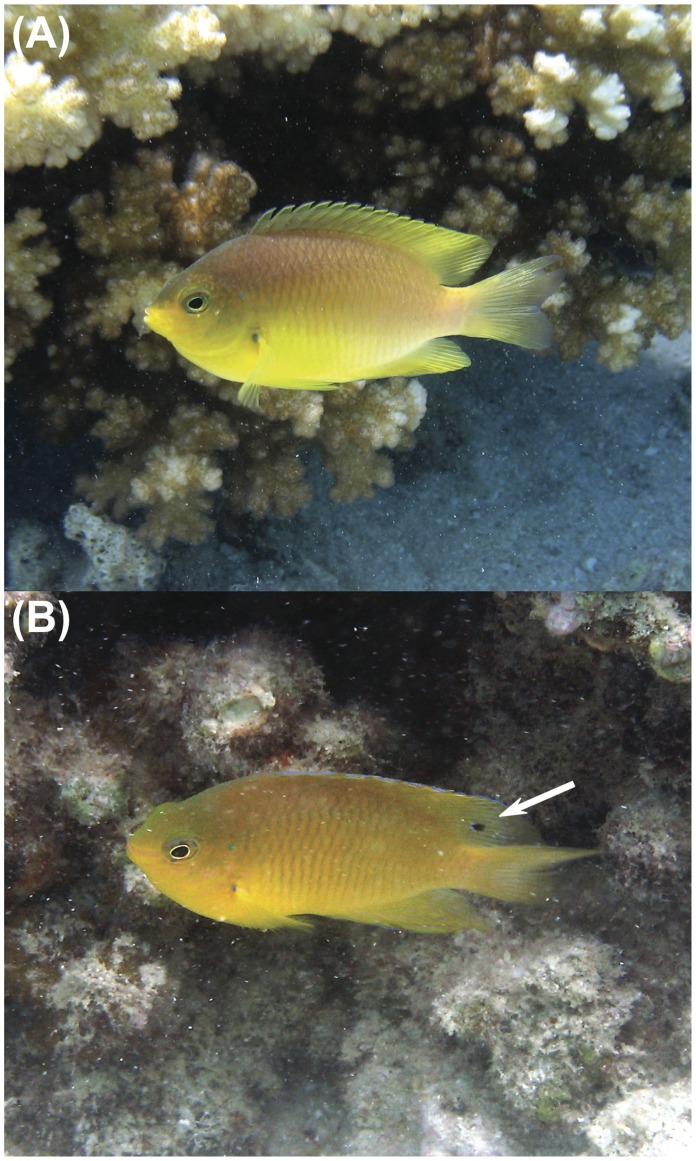
The damselfish *P. amboinensis*. (a) Sexually mature male without the eyespot and (b) sexually mature male still retaining the typically juvenile eyespot on the dorsal fin [indicated by the white arrow].

While most *P. amboinensis* exhibiting an eyespot on the dorsal fin ranged from newly settled recruits to older (but still) immature individuals, a significant 25% of all individuals with eyespots were sexually mature males (Figure 1). Given that this species forms social groups that contain individuals of all ages, where juvenile and mature females are loosely centred around a dominant male (lacking eyespot) and where growth rates and time to sexual maturity of juveniles is under social control [18], it is intriguing to find individuals that have matured into males while retaining a juvenile trait, such as the eyespot.

**Figure 2:**
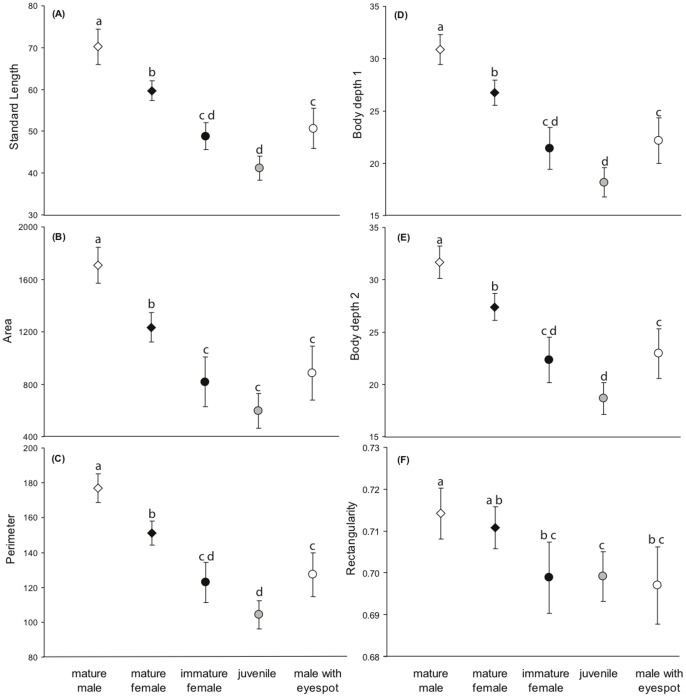
Comparison of the body dimensions of *P. amboinensis* belonging to different age and gender groups without eyespot (*diamonds*; N-values = 23 males & 36 females) and with eyespot (*circles*; N-values = 14 males, 16 females, 24 juveniles). Means ±95% CI. Letters indicate significant differences based on post-hoc Tukey’s HSD tests (i.e. fish groups exhibiting no significant difference in a given trait are indicated by the same letter).

**Figure 3:**
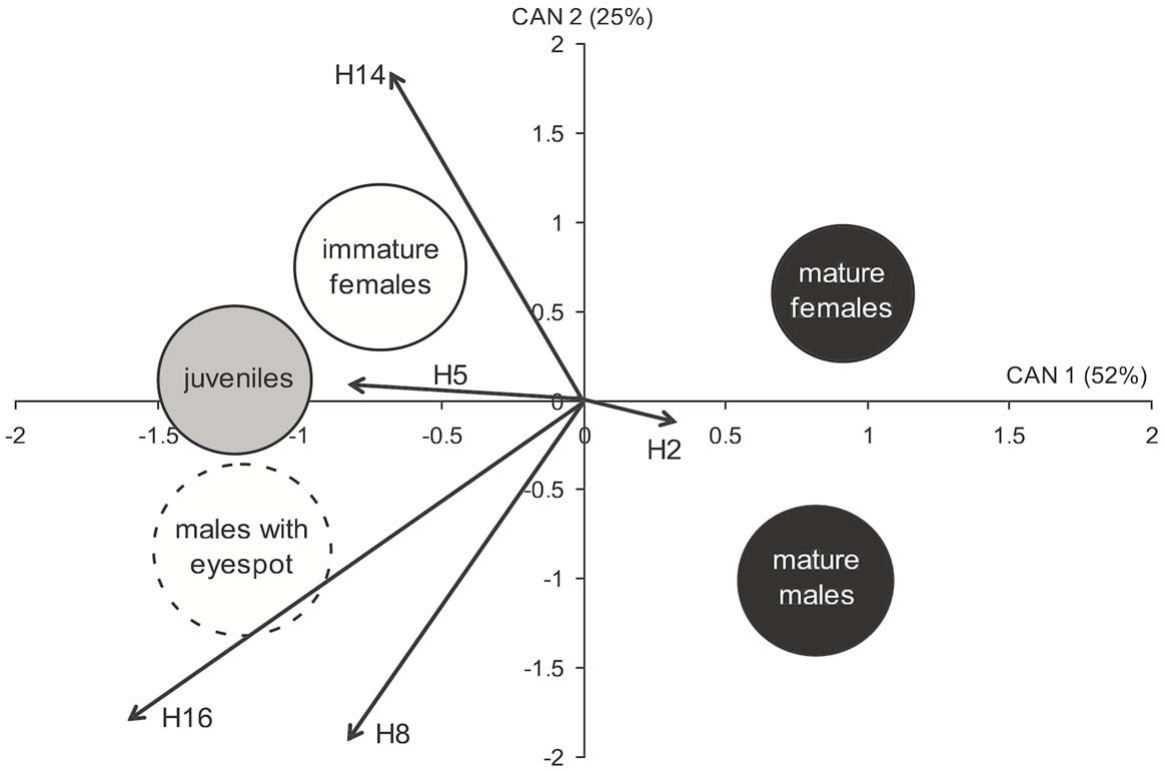
Comparison of the body shape of *P. amboinensis* belonging to different age and gender groups. Results of a canonical discriminant analysis are displayed with treatment centroids and 95% confidence clouds plotted together with the direction and importance (as indicated by the length of the vector) of trends in statistically significant shape descriptors.

Using Fast Fourier shape analyses, we found significant differences in the body dimensions and shape of *P. amboinensis* depending on their maturation status and the presence/absence of an eyespot on the dorsal fin. Unsurprisingly, mature non-spotted individuals were significantly bigger (Figure 2A–E) and more rectangular than immature ones (Figure 2F), with mature males being the largest and having the most rectangular body (one-way ANOVA for SL, F_4,98_ = 45.98, p<0.001; A, F_4,98_ = 37.99, p<0.001; DB_1_, F_4,98_ = 46.03, p<0.001; DB_2,_ F_4,98_ = 40.25, p<0.001; one-way ANOVA, F_4,98_ = 5.58, p<0.001). It was interesting, however, to find that the body shape of sexually mature but eye-spotted males more closely resembled that of juveniles and immature females than that of mature males. We found significant differences in the overall body shape of fish from the different groups (MANOVA Wilks λ, F_96,299_ = 1.66, p<0.001). Much of the variation among groups (52%) was due to coarse (i.e. low level harmonics H_2_ and H_5_) differences between the body shape of adults (with no eyespots) and that of individuals with eyespots, including mature males (canonical variate 1, Figure 3). Males with eyespots were located well away from both mature males and females with no eyespot along canonical variate 1 but occupied a similar position to males with no eyespots along the second variate (accounting for 25% of the overall variation). The arrangement of the 5 groups along this second axis suggests that those sexually mature individuals who have retained their dorsal eyespot have a “male” body shape at the finer scale (i.e. higher level harmonics, H_8_, H_14_, H_16_), albeit camouflaged in juvenile/immature female shape.

It is clear that becoming a dominant male in a social system full of mature females is reproductively advantageous. Yet within this social setting, what is the advantage of maturing into a male without acquiring a dominant social status, but instead retaining a juvenile trait? Visually prominent and colourful “ornaments” are generally costly and hence paraded only by individuals of superior quality or condition as signals of mate quality in a female choice context. For example, eyespots on butterfly wings have been previously reported to play an important role in intraspecific interactions, where females may exercise their preference for males with larger/darker eyespots [14], [31]. In *P. amboinensis*, however, eyespots are very affordable [11] and hence easily maintained and displayed by non-dominant males, strongly discounting the possibility that their message is directed to females in order to sway their mating preferences.

Together with their overall body morphology, the occurrence of reproductively active males with eyespots supports the idea that eyespots in animals may indeed have multiple roles. Although it has been previously recognized that eyespots can have multiple roles with at times conflicting functions, such as sexual selection (and specifically female choice) and predator avoidance [32], this is the first time that these markings are found to be important for their deceptive function in the context of intrasexual (male-male) competition. Moreover, these findings suggest that the functional significance of these markings in *P. amboinensis* switches within a lifetime from being a juvenile advertisement to a deceptive signal of age and non-breeding status. Clearly, the hypothesis that the eyespot is a deceptive signal would benefit from examining if males with eyespots have large gonads compared to males without eyespots and whether these spotted males are indeed successful in extra pair or group spawning activities.

In systems where male reproductive success is shaped by male-male competition and aggression, individuals have been observed to adopt alternative reproductive behaviours [33]. For example, males may parasitize the territory of dominant male conspecifics when they are competitively inferior, possibly due to their smaller size. These parasitic males may try to steal fertilizations by rushing into the nest guarded by their large, aggressive territorial counterparts just at the moment of egg deposition (i.e. sneakers). However, when a display of typical male behaviour is associated with a low probability of successful competitive outcomes (and hence high fitness costs) these non-dominant males may adopt a deceptive appearance or behaviour approach enabling them, for example, to follow the female to the nest in the guise of juveniles (i.e. juvenile mimics) to fertilize freshly laid eggs before the resident male can do so. Alternatively, they may mimic the behaviour and appearance of reproductive females (i.e. female mimics) in order to gain access to reproductive females attracted to the nests of dominant males. Either way, non-dominant individuals with inherently smaller body sizes are likely to be more reproductively successful by adopting one of these strategies when competing against dominant males than simply accepting their fate as overtly inferior males (i.e. making the best of a bad situation).

**Figure 4:**
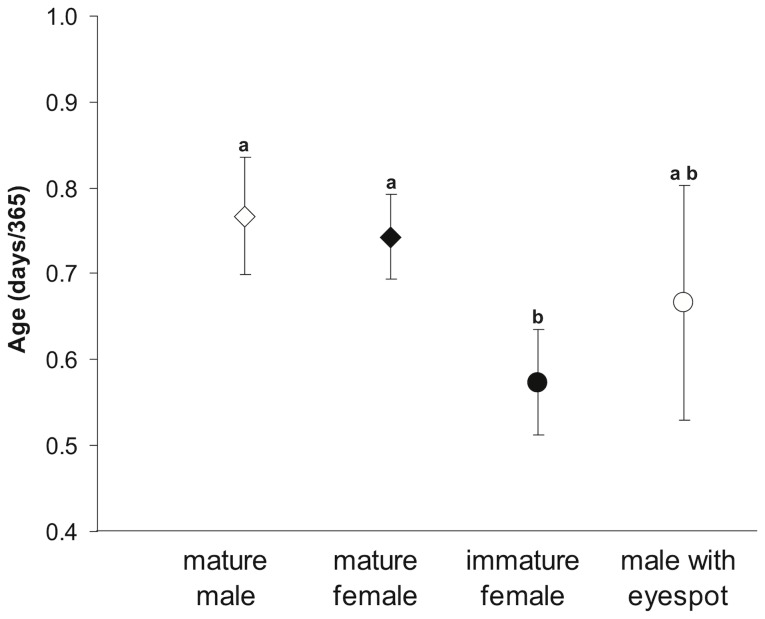
Mean age in days of *P. amboinensis* belonging to different age and gender groups without eyespot (*diamonds*; N-values = 21 males & 36 females) and with eyespot (*circles*; N-values = 9 males & 14 females). Error bars indicate 95% CI. Letters indicate significant differences based on post-hoc Tukey’s HSD tests.

Many species exhibit age-dependent variation in their reproductive tactics by sneaking when young and becoming parentals when older [33] and hence, sneaking is generally considered to be a conditional strategy based on body size. Mimicry, and specifically female mimicry on the other hand, requires gonadal development and gamete production to be decoupled from the female phenotype, and hence it may underscore some genetically embedded behavioural pathways [34]. In principle, mimicry would allow young males to more easily bypass the territorial defences of older males and gain access deceptively to territories and females. In the attempt to elucidate which strategy best describes the study system, we aged a total of 80 *P. amboinensis* with and without eyespots. Interestingly, eye-spotted males were not significantly younger than mature individuals who have lost their eyespots (one-way ANOVA, F_3,75_ = 5.41, p<0.002, where [F without eyespot = M without eyespot] ≠ F with eyespot; M with eyespot = all groups; Figure 4), suggesting that these individuals are not adopting an age-dependent conditional sneaking strategy (i.e. sneaking when young and waiting to become parentals when older). However, it could be that sneaking is conditional with respect to some other trait than age (e.g. size). Our results show that these individuals were clearly older for a given body size (ANCOVA, F_3,74_ = 3.02, p<0.05), indicating that they may invest more heavily in reproduction instead of growth. In addition, mature eye-spotted males reached maturation and changed sex at an earlier age (at 0.6 yr defined as the median age of the individuals in this group) than any of the mature fish without eyespots (over 9 months old for both sexes), reinforcing the idea that these individuals may be pre-disposed towards becoming mimics, while the others delay maturation in favour of growth to then become parentals [35].

So, do conditions experienced early in life determine which tactic an individual should choose? In some species, reproductive behaviours are fixed through genetic pathways (i.e. genetic polymorphism [36]). In others, processes operating prior to spawning such as non-genetic parental effects, may pre-determine which fish will later become a dominant male in a group [37]. Alternatively, it is equally possible that their early juvenile environment and individual personalities (e.g. shy/bold and aggressive/subordinate; [38]) shape their future reproductive life history strategy [39]. For instance, individuals recruiting late in the season that settle in a reef environment already populated by slightly older, larger and bolder juveniles from previous cohorts, may be more likely to experience aggressive behaviours and such social interactions may promote the development of a subordinate ‘personality’ with carry-over effects on their adult life. Of course, we acknowledge that these are currently speculative interpretations of our results.

We now know that eyespots do not function as an anti-predatory device in this species, but are instead honest signals of juvenile status and hence interpreted by con-specifics as subordinate and non-threatening [11]. Here we have shown that while most individuals lose their eyespots on maturation, some retain them into adulthood, that this retention is gender-specific (suggesting some reproductive advantage) and that it is further enhanced by eye-spotted males having a closer resemblance to females than the males they actually are.

Clearly, the next logical step is to test a number of hypotheses using experimental and manipulative approaches so that we can fully appreciate and understand the range of functional roles eyespots have in animals.
